# An attention based hybrid approach using CNN and BiLSTM for improved skin lesion classification

**DOI:** 10.1038/s41598-025-00025-2

**Published:** 2025-05-05

**Authors:** Ayesha Shaik, Shivanya Shomir Dutta, Ishaan Milind Sawant, Shreyas Kumar, Ananthakrishnan Balasundaram, Kanjar De

**Affiliations:** 1 Centre for Cyber Physical Systems, Vellore Institute of Technology (VIT), Chennai, 600127 India; 2 School of Computer Science and Engineering, Vellore Institute of Technology (VIT), Chennai, 600127 India; 3 Video Coding Systems, Fraunhofer Heinrich-Hertz-Institut, Berlin, Germany

**Keywords:** CNN, BiLSTM, Spatial attention, Channel attention, Temporal attention, Skin lesion classification., Biomedical engineering, Skin diseases

## Abstract

Skin lesions remain a significant global health issue, with their incidence rising steadily over the past few years. Early and accurate detection is crucial for effective treatment and improving patient outcomes. This work explores the integration of advanced Convolutional Neural Networks (CNNs) with Bidirectional Long Short Term Memory (BiLSTM) enhanced by spatial, channel, and temporal attention mechanisms to improve the classification of skin lesions. The hybrid model is trained to distinguish between various skin lesions with high precision. Among the models evaluated, the CNN (original architecture) with BiLSTM and attention mechanisms model achieved the highest performance, with an accuracy of 92.73%, precision of 92.84%, F1 score of 92.70%, recall of 92.73%, Jaccard Index (JAC) of 87.08%, Dice Coefficient (DIC) of 92.70%, and Matthews Correlation Coefficient (MCC) of 91.55%. The proposed model was compared to other configurations, including CNN with Gated Recurrent Units (GRU) and attention mechanisms, CNN with LSTM and attention mechanisms, CNN with BiGRU and attention mechanisms, CNN with BiLSTM, CNN with LSTM, CNN with BiGRU, CNN with GRU, standalone CNN, InceptionV3, Visual Geometry Group-16 (VGG16), and Xception, to highlight the efficacy of the proposed approach. This research aims to empower healthcare professionals by providing a robust diagnostic tool that enhances accuracy and supports proactive management strategies. The model’s ability to analyze high-resolution images and capture complex features of skin lesions promises significant advancements in early detection and personalized treatment. This work not only seeks to advance the technological capabilities in skin lesion diagnostics but also aims to mitigate the disease’s impact through timely interventions and improved healthcare outcomes, ultimately enhancing public health resilience on a global scale.

## Introduction

Skin lesions^1,2^ are a pervasive health concern worldwide, characterized by abnormal growths on the skin that can lead to severe health consequences if not detected early. The incidence of skin lesions has been steadily rising in recent years, highlighting the critical need for effective detection and treatment strategies. Early and accurate diagnosis is essential for enabling timely intervention and personalized treatment plans, which in turn improve patient outcomes and reduce the overall healthcare burden. Machine learning (ML), particularly through advanced Convolutional Neural Network (CNN) techniques, has emerged as a promising tool in medical imaging and diagnostics. These models have the ability to analyze high-resolution images of skin lesions, distinguishing between benign and malignant tissues with remarkable accuracy. This capability is vital for early detection and holds the potential to revolutionize the way skin lesions are diagnosed and treated, ensuring that patients receive the care they need at the earliest possible stage.

A recent research^3^ indicates a significant expansion in the global dataset for skin lesion images used for training ML models. Datasets from repositories such as the International Skin Imaging Collaboration (ISIC) and DermNet NZ have provided researchers with a wide array of skin lesion images, covering diverse types and pathological conditions. Leveraging these comprehensive datasets, researchers have developed sophisticated CNN architectures, such as VGG-16 and ResNet, which have shown outstanding performance in classification tasks.

This work aims to advance the field of skin lesion classification by leveraging a hybrid model that integrates CNN with Bidirectional Long Short Term Memory (BiLSTM), augmented with spatial, channel, and temporal attention mechanisms. This innovative approach is designed to capture the complex features and variations in skin lesion images more effectively than traditional models. By elucidating the nuances of different skin lesion types, this research endeavors to empower healthcare professionals with robust tools for accurate diagnosis and proactive management strategies. Ultimately, this research holds promise in mitigating the impact of skin lesions through timely intervention and personalized care. By improving diagnostic accuracy and enabling more effective treatment plans, it aims to enhance individual health outcomes and bolster public health resilience on a global scale. Integrating advanced machine learning techniques with comprehensive datasets represents a significant step forward in the fight against skin lesions, offering hope for better management and control of this widespread health issue.

The motivation to develop a model that combines CNNs with BiLSTM networks and multiple attention mechanisms stems from the complex nature of the dermatological diagnosis. Skin lesions can vary greatly in appearance, making it essential to capture both spatial and temporal information. CNNs excel at identifying detailed spatial features, while RNNs, particularly BiLSTMs, are adept at handling temporal dependencies. BiLSTMs enable the model to consider the sequence of features within each image, preserving contextual information across spatial dimensions. Integrating these architectures allows for a more comprehensive analysis of skin lesions, capturing their intricate details and progression. Additionally, attention mechanisms enhance the model’s interpretability by highlighting the most relevant regions and features, providing clinicians with insights into the decision making process. This approach seeks to reconcile the high accuracy of AI models with the necessity for transparency and trust in medical applications, ultimately enhancing diagnostic precision and fostering better clinical outcomes. The major contributions of this work are listed below.

- An integrated model using Convolutional Neural Networks (CNNs) with Bidirectional Long Short Term Memory (BiLSTM) enhanced by spatial, channel, and temporal attention mechanisms to improve the classification of skin lesions has been proposed.
- An exhaustive experimentation of various approaches along with conventional CNN was performed and reported using several performance metrics.
- The best performing model was validated with two more datasets^56,57^ and also with other contemporary works^17,25–27^.

The manuscript structure is organized into different sections to provide a unique approach towards skin lesion classification.

Sect “Related works” presents a review of the previous existing literature available on skin lesion classification and the unique approaches used previously. Sect “Proposed methodology” presents the methodology where first the reason for the proposed unique approach is described, then an overview of the different Deep Learning (DL) techniques and attention mechanisms that were used to form the proposed model, its counter models, and other models for comparison. After that, it provides the description of the proposed model, the training process, and the metrics that were used to evaluate it. Sect “Experimental results and discussion”, provides detailed results and discussions. Finally, Sect “Conclusion and future work” concludes the study and outlines future directions.

## Related works

The rapid advancement of deep learning technologies has significantly impacted the field of dermatological imaging and skin lesion detection. Various studies have explored innovative methodologies, leveraging advanced DL models to enhance diagnostic accuracy and support medical training. This review synthesizes key findings from recent research, focusing on the use of CNNs and Transfer Learning (TL) models to improve the detection and classification of skin lesions. Chang et al. utilized the InceptionResNetV2 model, developed by Google, in conjunction with a custom CNN to enhance image resolution and analysis within the HAM10000 database. This system was designed to assist dermatological residents in recognizing imaging characteristics of skin lesions outside standard training hours. By allowing residents to upload photos of skin lesions for real-time AI analysis, the system provides immediate probability assessments of various skin diseases. This feedback mechanism aims to expedite the learning curve for residents, enriching their diagnostic skills and experience with skin conditions^4^. Similarly, Khattar, Kaur, and Gupta combined a self-developed CNN with Google’s InceptionResNetV2 to analyze and classify images from the HAM10000 dataset, focusing on improving the training process for dermatology residents. They tested multiple neural network models, finding that DenseNet201 achieved the highest classification accuracy of 93.24%, with an AUC of 0.932^5^.

Shete et al. explored integrating InceptionResNetV2 with a custom CNN to classify skin lesions, achieving a weighted average precision, recall, f1 score of 0.88, 0.74 and 0.77 respectively, with ResNet achieving a classification accuracy of 90.51%^6^. In another study, Moataz et al. fine-tuned the Xception model for skin lesion image classification, achieving a training accuracy of 99.6% and validation accuracy of 96%, highlighting the potential of transfer learning in skin lesion classification^7^. Similarly, Garg, Maheshwari, and Shukla employed InceptionResNetV2 alongside a custom CNN, achieving similar high training and validation accuracies, underscoring the model’s effectiveness in classifying skin lesions^8^.

Shehzad et al. further demonstrated the efficacy of combining InceptionResNetV2 with a custom CNN, achieving a training accuracy of 99.6% and validation accuracy of 96%, showcasing the potential of advanced CNN architectures and transfer learning techniques in improving the diagnostic process for skin lesions^9^. Claret, Dharmian, and Manokar introduced a method combining CNNs with discrete wavelet transformation (DWT) to enhance early diagnosis of skin lesions. Their approach used DWT to extract relevant features from skin lesion images, processed by the CNN model, achieving a sensitivity of 94% and a specificity of 91%, surpassing traditional methods^10^. Satapathy et al. employed a Capsule Network (CapsNet) for skin lesions classification using the HAM10000 dataset. The CapsNet model, designed to capture spatial hierarchies and positional information more effectively than traditional CNNs, achieved an overall average precision, recall, and F1-score of 0.94, 0.91, and 0.91, respectively^11^. Çevik and Zengin introduced a deep CNN model leveraging the VGGNET-16 architecture to classify dermoscopic images, achieving a validation accuracy of 85.62%^12^. Lastly, Ali et al. proposed a novel DCNN model with unique modifications, achieving a classification accuracy of 97.56%, precision of 96.85%, recall of 97.12%, and an F1-score of 96.98%, demonstrating its superiority over existing approaches^13^.

The work by Marwan Ali Albahar^14^ examines CNNs for skin lesion classification, crucial for early skin lesion detection. A novel regularizer is introduced to prevent overfitting and enhance accuracy. However, the paper lacks detailed information on the regularizer’s mechanics, the dataset, and comparative analysis with other methods, which limits a comprehensive understanding of its contributions. In another study focusing on facial skin diseases, Wu et al.^15^ explored the application of various CNN architectures using the Xiangya-Derm dataset, comprising 2656 clinical images across six distinct conditions. Their investigation highlighted the efficacy of transfer learning, with Inception-ResNet-v2 demonstrating superior performance in classifying skin diseases. However, challenges such as dataset imbalance and clinical overlap among disease categories underscored the need for improved data management strategies and refined model training approaches to mitigate classification inaccuracies.

Kassem et al.^16^ addressed the classification of skin lesions using the ISIC 2019 dataset, renowned for its complexity and large-scale diversity among different lesion types. Their approach involved enhancing the GoogleNet architecture through additional filters, achieving notable accuracy across eight distinct lesion classes. Despite initial challenges related to dataset imbalance, the study demonstrated significant improvements through rigorous data augmentation techniques, thereby enhancing the model’s capability for accurate diagnosis and detection of outlier lesions. Ahmad et al.^17^ introduced a novel framework for skin disease classification by integrating triplet loss functions with deep CNN models, specifically ResNet152 and InceptionResNet-V2. This approach aimed to enhance the discriminative feature learning capabilities of the models, resulting in commendable accuracy levels. Nonetheless, challenges associated with dataset specificity and the computational demands of fine-tuning deep networks were acknowledged as potential limitations, highlighting the need for further advancements in algorithmic efficiency and model generalizability.

Anjum et al.^18^ propose a comprehensive three-phase framework utilizing models like YOLOv2, ResNet-18, and Ant Colony Optimization achieves high accuracy in detecting skin lesions. and classification, although the approach demands substantial computational resources. Goyal et al.^19^ reviews various DL methods, highlighting ensemble techniques such as DeeplabV3 + and Mask R-CNN, which combine semantic and instance segmentation to handle noisy annotations and improve robustness, but these methods are computationally intensive and require meticulous preprocessing. Lastly, Natasha Nigar et al.^20^ emphasize the importance of explainability in their approach, integrating ResNet-18 with the LIME framework to provide visual justifications for predictions, thereby enhancing trustworthiness in clinical settings. However, the reliance on a single dataset and occasional inconsistencies in LIME’s explanations pose limitations. Together, these studies underscore the potential and challenges of deploying DL models in dermatological diagnostics, balancing accuracy, interpretability, and computational demands.

Bian et al.^21^ explored the efficacy of Multi-View Filtered Transfer Learning (MFTL) using datasets from ISIC 2017 and HAM10000, showcasing significant improvements in model performance through selective sample distillation via Wasserstein distance. However, the study noted computational complexities and preprocessing challenges as potential barriers to widespread implementation. Adegun and Viriri^22^ introduced an innovative (Fully Convolutional Network) FCN-based DenseNet framework for automated skin lesion detection and classification, emphasizing computational efficiency and accuracy enhancement through optimized feature processing. Despite achieving high performance metrics on the HAM10000 dataset, limitations related to dataset scarcity and computational resource demands were highlighted as areas necessitating further exploration and refinement.

Naeem et al.^23^ conducted a comprehensive review of CNN-based approaches for melanoma detection, emphasizing the critical role of diverse and expansive datasets in improving model robustness and accuracy. Challenges related to dataset diversity, computational efficiency, and model interpretability were identified as crucial avenues for future research to enhance the clinical applicability and reliability of AI-driven diagnostic tools in dermatology.

Thurnhofer-Hemsi et al.^24^ proposed an ensemble CNN approach with test-time shifting techniques for skin lesion classification, addressing dataset imbalance and achieving superior performance metrics on the challenging HAM10000 dataset. Despite the advancements, the study acknowledged computational complexities associated with ensemble learning and the need for further research to optimize model scalability and generalizability across diverse clinical scenarios.

Another^25^ study proposes an integrated framework for automated skin lesion diagnosis using dermoscopy images, combining lesion boundary segmentation with deep learning-based classification. The segmentation stage, performed by a full-resolution convolutional network (FrCN), enhances feature extraction for classifiers like Inception-v3, ResNet-50, Inception-ResNet-v2, and DenseNet-201. Evaluated on ISIC datasets (2016, 2017, 2018), the framework improved classification performance, with Inception-ResNet-v2 achieving notable F1-score gains for benign and malignant cases. Weighted accuracies reached up to 89.28% across datasets. This highlights the value of integrating segmentation and classification for accurate skin lesion analysis.

^17^ addresses the growing prevalence of skin diseases, which pose psychological and physical risks, including skin cancer. Due to limitations in visual resolution and the subjectivity of manual diagnosis, a computer-aided diagnostic framework is proposed using ResNet152 and InceptionResNet-V2 with a triplet loss function. The framework maps input images into Euclidean space to compute L-2 distances for learning discriminative features. These features are then used to classify skin disease images effectively. The dataset consists of human facial skin disease images collected from a hospital in Wuhan, China. Experimental results demonstrate the framework’s ability to improve the accuracy and efficiency of skin disease diagnosis.

^26^ addresses the challenge of training deep learning models with limited data by introducing the Predict-Evaluate-Correct K-fold (PECK) algorithm, which trains deep ensembles on 153 non-dermoscopic lesion images. PECK combines convolutional neural networks, support vector machines, and random forests, using hierarchical learning to iteratively correct prediction errors. Additionally, the SCIDOG segmentation algorithm is proposed to detect lesion contours in noisy, non-dermoscopic images without relying on data-intensive training. SCIDOG enables precise lesion feature extraction, which, combined with PECK, enhances melanoma and benign nevi diagnosis on the MED-NODE dataset. The proposed methods achieve superior diagnostic performance using 10-fold cross-validation, surpassing prior state-of-the-art techniques.

^27^ presents a fully automated method for classifying skin lesions from dermoscopic images, addressing the challenge of distinguishing malignant melanomas from benign lesions. The approach ensembles deep features from multiple pretrained convolutional neural networks (CNNs), fine-tuned on limited dermoscopic data, with a support vector machine classifier. Prediction probabilities from various models are fused to enhance classification accuracy. On the ISIC 2017 dataset, the method achieves an AUC of 87.3% for melanoma and 95.5% for seborrheic keratosis, surpassing top-ranked methods in simplicity and performance. This demonstrates a reliable and robust solution for dermoscopic image analysis and skin lesion diagnosis.

In summary, recent studies underscore significant advancements in skin lesion classification through the integration of diverse datasets, advanced model architectures, and innovative techniques such as transfer learning and attention mechanisms. Despite these advancements, challenges related to dataset diversity, computational intensity, and model interpretability remain critical focal points for future research aimed at enhancing the clinical utility and reliability of AI-driven diagnostic tools in dermatology. Table 1 lists the closely related contemporary works in this area.

**Table 1:** Literature survey of comtemporary works.

Reference number	Proposed model	Benefits	Challenges
^4^	InceptionResNetV2 + Custom CNN	Immediate probability assessments of skin diseases; expedited learning curve for residents	Integration and optimization of AI program; validation in clinical settings
^5^	InceptionResNetV2 + Custom CNN (DenseNet201)	High classification accuracy; effective training for dermatological residents	Model complexity; computational resources required
^6^	InceptionResNetV2 + Custom CNN	Improved pixel calculations; high accuracy	Dataset diversity; generalizability to diverse skin types
^7^	Fine-tuned Xception	High training and validation accuracy; robust performance	Data augmentation challenges; class imbalance
^8^	InceptionResNetV2 + Custom CNN	High accuracy; practical learning tool	Model interpretability; deployment in clinical environments
^9^	InceptionResNetV2 + Custom CNN	Superior performance; ensemble model	Training time; overfitting concerns
^10^	CNN + Discrete Wavelet Transformation	Improved accuracy; feature extraction	Complexity of feature extraction; integration with existing systems
^11^	Capsule Network (CapsNet)	High precision, recall, F1-score; spatial hierarchy capture	Limited research; interpretability of Capsule Networks
^12^	VGGNET-16	High validation accuracy; effective dermoscopic image classification	Model depth; computational efficiency
^13^	Deep CNN (DCNN)	High accuracy; superior performance in multi-class classification	Model complexity; scalability to larger datasets
^14^	CNN with novel regularizer	Introduces a unique regularizer to enhance generalization and classification accuracy	Lack of detailed regularizer formulation, insufficient discussion on dataset specifics, potential computational complexity
^15^	ResNet-50, Inception-v3, DenseNet121, Xception, Inception-ResNet-v2	Achieves high performance in facial skin disease classification, benefits from transfer learning	Imbalanced dataset distribution, challenges in distinguishing clinically similar conditions, difficulties in model interpretability
^16^	GoogleNet modified for skin lesion classification	Demonstrates high accuracy in classification, including detection of unknown lesion types	Imbalance in dataset classes, variability in lesion appearance, issues with dermoscopic artifacts
^17^	ResNet152, InceptionResNet-V2 with triplet loss function	Implements triplet loss for improved feature learning and classification accuracy	Dependency on specific dataset, demands for computational resources for fine-tuning deep networks
^18^	YOLOv2, 3D-semantic segmentation model, ResNet-18, ACO, SVM, Naive Bayes	Integrates comprehensive framework for lesion localization, segmentation, and classification	High computational requirements, dataset imbalance affecting performance, extensive data augmentation needs
^19^	Ensemble methods (DeeplabV3+, Mask R-CNN)	Combines semantic and instance segmentation for improved accuracy and robustness	Computational complexity due to deep architectures, challenges in pre-processing and post-processing steps
^20^	ResNet-18 with LIME framework	Provides high accuracy with interpretability through visual explanations	Relies heavily on a single dataset, occasional inconsistency in LIME explanations
^21^	Multi-View Filtered Transfer Learning	Enhances discriminatory power using multiple views and selective sample distillation	High computational demands, requires extensive preprocessing and data augmentation
^22^	FCN for segmentation, DenseNet-based FCN for classification	Achieves high accuracy while reducing network complexity and computational resources	Limited availability of labelled data, challenges in multi-scale feature processing
^23^	Review paper on CNN-based classifiers for melanoma detection	Provides comprehensive overview and identifies emerging trends in melanoma detection	Limited by small and non-diverse datasets, high computational requirements for robust model training
^24^	Ensemble of MobileNetV2 and GoogLeNet with regularly spaced shifting technique	Improves accuracy through ensemble learning and innovative shifting technique	Computational intensity due to ensemble approach, dataset imbalance affecting model performance
^25^	Integrated framework combining lesion segmentation (FrCN) with deep learning-based classification (e.g., Inception-v3, ResNet-50, DenseNet-201).	Enhanced feature extraction through segmentation. Improved F1-score and weighted accuracy (up to 89.28%). Effective across multiple ISIC datasets	High computational cost due to integration of segmentation and classification stages.
^17^	Diagnostic framework using ResNet152 and InceptionResNet-V2 with triplet loss function.	Effective mapping of features in Euclidean space. Improved accuracy in facial skin disease diagnosis. Addressed challenges in manual diagnosis.	Dataset limited to facial skin disease images. Model generalizability for other types of skin diseases.
^26^	Predict-Evaluate-Correct K-fold (PECK) algorithm with SCIDOG segmentation algorithm.	Successfully trained models with limited data. Enhanced lesion feature extraction from noisy images. Superior diagnostic performance on MED-NODE dataset.	Limited evaluation dataset (153 images). Potential difficulty scaling to larger datasets.
^27^	Fully automated classification using ensembled deep features from pretrained CNNs fused with support vector machines.	High AUC for melanoma (87.3%) and seborrheic keratosis (95.5%). Simpler yet effective compared to top-ranked methods in ISIC 2017 challenge.	Limited by the availability of annotated dermoscopic images. Reliance on pretrained models for fine-tuning.

## Proposed methodology

In the proposed methodology, a custom CNN architecture is employed in combination with BiLSTM units and Attention Mechanisms for skin lesion classification. The rationale behind using CNNs in conjunction with RNNs lies in their complementary strengths: CNNs excel at capturing intricate spatial features in images, while RNNs, particularly BiLSTM units, are adept at analyzing sequential data and temporal patterns. This combination ensures that both spatial and sequential aspects of the data are effectively utilized, leading to more accurate and robust classification. According to the literature survey, there were no prior experiments that combined CNN and RNN for this specific application. Understanding the strengths of CNN, RNN, and Attention Mechanisms in tandem is crucial as it highlights the potential of these advanced techniques to revolutionize medical diagnostics.

Additionally, attention mechanisms are incorporated to further enhance the performance of the CNN-RNN combination. Attention mechanisms allow the model to focus on the most relevant parts of the input data, ensuring that critical features are given more weight during the analysis. This not only improves the model’s accuracy but also its interpretability, making it easier for clinicians to understand and trust the model’s predictions. The techniques implemented in the proposed model, along with a comparison against other pretrained models and their respective counterparts, are described in detail below. This comparison underscores the superiority of the proposed model in terms of accuracy, precision, recall, and overall robustness, validating the effectiveness of the proposed work.

### Implementation of deep learning techniques

To capture sequential dependencies in the data, Recurrent Neural Networks (RNNs) like Long Short-Term Memory (LSTM) units, Gated Recurrent Units (GRUs), Bidirectional LSTM, and Bidirectional GRU are employed. These RNNs allow the proposed model to maintain and transfer information from previous steps of convolution layers, enhancing its ability to understand sequential patterns.

The proposed approach integrates RNNs with a custom CNN and attention mechanisms, forming a robust hybrid architecture. The proposed model’s performance is evaluated against several other models, including pre-trained networks like ResNet-50, InceptionV3, and VGG16, as well as configurations using only custom CNNs and custom CNNs combined with RNNs. The proposed model, which merges a custom CNN with RNNs and attention mechanisms, proved to be the most effective among the tested configurations.

#### Custom CNN model

The custom Convolutional Neural Network (CNN) is designed to efficiently extract spatial features from input images. The model starts with an input layer that accepts images of size (224, 224, 3), corresponding to 224 × 224 pixels with 3 color channels (RGB). It includes four convolutional layers: the first layer has 32 filters of size (3, 3) with ReLU activation and ‘same’ padding, followed by the second layer with 64 filters, the third with 128 filters, and the fourth with 256 filters, all using the same kernel size and activation function. Each convolutional layer is followed by a MaxPooling2D layer with a pool size of (2, 2), which reduces the spatial dimensions by half, effectively downsampling the feature maps while preserving essential features.

After the final convolutional and pooling layers, the output is flattened and passed through a fully connected (dense) layer with 512 units and ReLU activation. This dense layer combines the features learned by the convolutional layers into a more abstract representation. To prevent overfitting, a Dropout layer with a 0.5 dropout rate is included, randomly setting 50% of the input units to zero at each update during training, helping the model generalize better. The final layer is a dense layer with units equal to the number of classes, using a softmax activation function. This layer provides the probability distribution over the classes for each input image, making it suitable for classification tasks.

#### Recurrent neural network (RNN)

RNNs^28^ are a type of neural network designed to handle sequential data by capturing temporal dependencies. In this section, four widely-used types of RNNs: LSTM, GRU, BiLSTM and BiGRU are discussed. But since the dataset comprises of images the following RNN techniques are only used in combination of the custom CNN model. These models created with the combination of the custom CNN and RNN are used for demonstrating the superiority of the proposed model.

##### Long short term memory (LSTM)

The LSTM^29^ is a specialized variant of RNNs, tailored for sequential data processing while circumventing the vanishing gradient problem often encountered in conventional RNNs. Its architecture is engineered to address this challenge. Every LSTM device has the basic components: a cell, an input gate, an output gate, and a forget gate. The cell is capable of retaining information across extended time steps, while the three gates regulate the flow of data into and out of the cell.

**Fig. 1:**
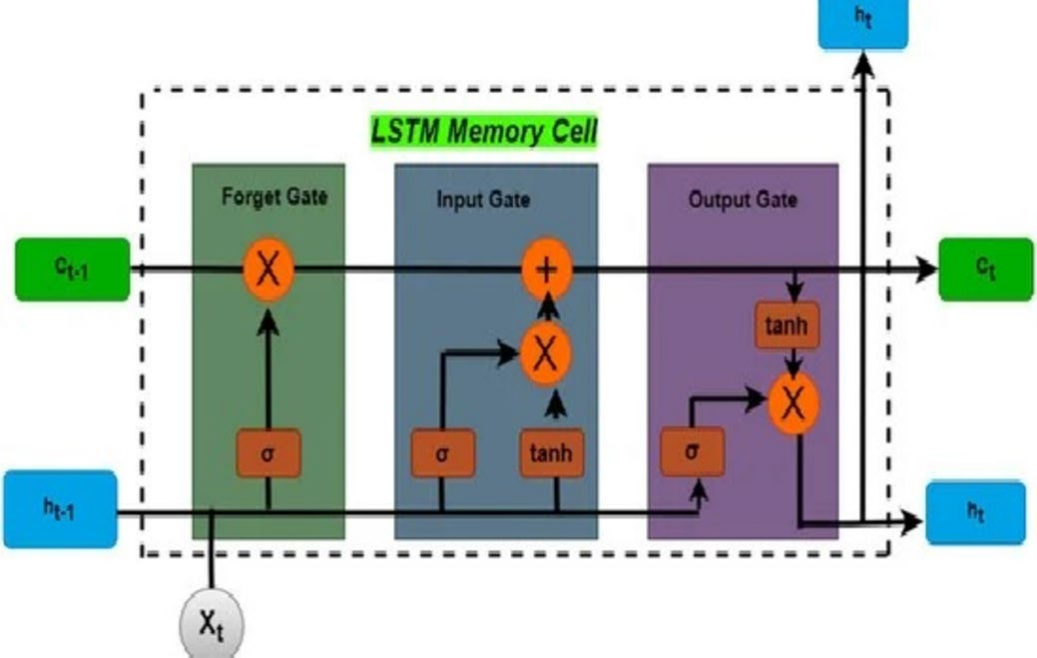
LSTM architecture^17^.

Specifically, the forget gate determines which information from the previous state should be discarded relative to the current input (ranging from 0 to 1), the input gate decides what new data should be stored, and the output gate determines which segments of the current state should be outputted, considering both the present and past states. These functional aspects are governed by computational Eqs. (1), (2), (3), (4), (5), (6) and is also depicted in the Fig. 1.

1 $$\:i{\:}_{t}=\:\sigma\:({x}_{t}{U}^{i}\:+\:{h}_{t-1}{W}^{i}\:)\:\:$$

2 $$\:{f}_{t}=\:\sigma\:({x}_{t}{U}^{f}\:\:+\:{h}_{t-1}{W}^{f}\:)\:\:$$

3 $$\:{o}_{t}\:=\:\sigma\:({x}_{t}{U}^{o}+\:{h}_{t-1}{W}^{o})\:\:$$

4 $$\:C\:=\:tanh({x}_{t}{U}^{g}+\:{h}_{t-1}{W}^{g}\:)\:\:$$

5 $$C_{t} ~ = ~\sigma \left( {f_{t} *~C_{{t - 1}} + ~i_{t} ~*~C_{t} ^{\sim } } \right)~$$

6 $$\:{h}_{t}=\:tanh\:\left({C}_{t}\right)\:*\:{o}_{t}t\:\:\:$$

##### Gated recurrent unit (GRU)

**Fig. 2:**
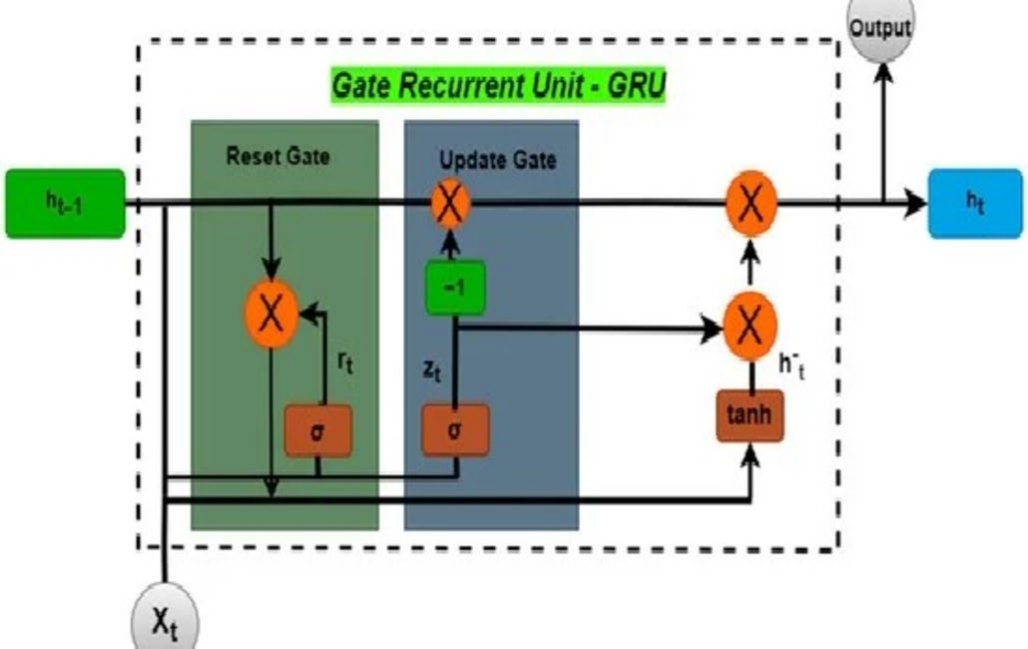
GRU architecture^17^.

The GRU^30^ is a specialized architecture within recurrent neural networks, designed to handle sequential data. It overcomes the limitations of traditional RNNs by incorporating gating mechanisms, which allow the network to selectively update and reset its hidden states. This feature enables GRUs to effectively capture long-range dependencies in the data. Unlike Long Short-Term Memory (LSTM) networks, which utilize three gates, GRUs use only two gates: an update gate and a reset gate. This streamlined design makes GRUs computationally efficient while retaining their effectiveness in tasks such as natural language processing and time series analysis. As a result, GRUs have become a versatile choice for various applications, balancing model complexity and performance in sequential data processing. These functional aspects are governed by computational Eqs. (7), (8), (9), (10) and is also depicted in Fig. 2.

7 $$\:{z}_{t}=\sigma\:\left({w}_{z}\cdot\:\left[{h}_{t-1},{x}_{t}\right]\right)$$

8 $$\:{r}_{t}=\sigma\:({w}_{r}.[{h}_{t-1},{x}_{t}\left]\right)$$

9 $$\hat{h}_{t} = \tanh \left( {w \cdot \left[ {r_{t} .h_{{t - 1}} ,x_{{\_t}} } \right]} \right)$$

10 $$\:{\varvec{h}}_{\varvec{t}}=(1-{\varvec{z}}_{\varvec{t}}.{\varvec{h}}_{\varvec{t}-1}+{\varvec{z}}_{\varvec{t}}.{\varvec{x}}_{\varvec{t}})\:$$

##### Bidirectional LSTM (BiLSTM)

Bidirectional LSTMs^31^ extend the traditional LSTM models by applying two LSTMs to the input data. In the first pass, an LSTM processes the input sequence in the forward direction (forward layer). In the second pass, the input sequence is reversed and fed into another LSTM (backward layer). This dual application of LSTMs enhances the model’s ability to learn long-term dependencies, thereby improving its accuracy.

##### Bidirectional GRU (BiGRU)

BiGRU^32^ represent an advanced RNN architecture designed to capture complex temporal dependencies in sequential data. The bidirectional nature allows for the integration of both past and future contextual information. Given an input sequence X, a BiGRU processes this input in two directions, computing forward hidden states and backward hidden states.

### Transfer learning

Several well-known pre-trained models, including ResNet-50^32^, VGG-16, and InceptionV3 are utilized, leveraging their learned representations and adapting them to the specific classification task. These pre-trained models were used as fixed feature extractors to compare with the proposed model. The input image was passed through the pre-trained model’s convolutional layers, and the resulting output features were used for classification.

Following the frozen layers, a dense layer with 1024 units is added to further process the extracted features, followed by a final classification layer, typically a softmax layer, to predict the class probabilities. This approach leverages the pre-trained weights without further fine-tuning, allowing the extracted features to capture meaningful information from the images. By comparing the performance of these transfer learning models with the proposed model, this work is aimed to evaluate the effectiveness and efficiency of the proposed approach in diagnosing skin lesions.

#### VGG-16 framework

VGG-16^34^ is a convolutional neural network (CNN) model developed by Karen Simonyan and Andrew Zisserman at the University of Oxford, primarily used for object detection and classification tasks. It enhances the capabilities of DL models with its 16 layers, each employing ReLU as the activation function, enabling effective feature extraction through nonlinear transformations of input data.

To optimize training efficiency, VGG-16 uses the Adam optimizer, an adaptive learning rate method that accelerates computation and reduces the need for extensive parameter tuning. In the output layer, the SoftMax activation function is employed to facilitate quick convergence and provide probability estimates for multi-class classification tasks.

#### InceptionV3 framework

InceptionV3^35^ is an advanced convolutional neural network (CNN) architecture renowned for its innovative use of inception modules. These modules utilize parallel convolutions with different kernel sizes, allowing the network to effectively capture multi-scale features. This approach enhances the model’s ability to extract intricate patterns and details from images, contributing to its high performance in diverse image classification tasks.

Having been pre-trained on extensive datasets, InceptionV3 showcases robust capabilities in handling complex visual data. Its architecture not only promotes efficient feature extraction through its inception modules but also incorporates advanced techniques to optimize training and enhance generalization. In various benchmarks and applications, InceptionV3 has consistently delivered impressive results, underscoring its reliability and effectiveness in the field of DL for image analysis and classification.

#### Xception framework

Xception^36^, short for “Extreme Inception,” is a deep learning model architecture introduced by François Chollet in 2017. This model aims to enhance computational efficiency by using a technique called split convolution. Split convolution divides the processes of spatial convolution and dimensionality reduction, which helps decrease the number of parameters and boosts efficiency. The Xception comprehends and extracts intricate feature representations from image data. Known for its high computational efficiency and strong performance in image recognition and classification tasks, Xception is frequently employed in image processing applications, especially on devices with limited resources.

### Implementation of attention mechanisms

#### Spatial attention

To extract the spatial information^37^ of different components of a building, the spatial relationship between features is leveraged to generate a spatial attention map, which emphasizes the spatial features and complements the channel attention. Initially, average pooling and max pooling operations are performed along the channel axis, resulting in two 2D maps: $\:{F}_{{S}_{avg}}\in\:{R}^{1\times\:H\times\:W}$and $\:{F}_{{S}_{max}}\:\in\:{R}^{1\times\:H\times\:W}$. These maps represent the average-pooling and max-pooling features across channels. The two maps are then concatenated along the channel dimension to create a combined feature descriptor. This concatenated feature descriptor is processed through a convolution layer with a kernel size of 7, padding set to ‘same,’ and a sigmoid activation function, resulting in the spatial attention map.

$$\:{M}_{S\:}\in\:\:{R}^{H\times\:W}$$

In this work, wherever spatial attention is used in combination with a model, the settings such as activation function, kernel size and padding have been kept the same. The spatial attention map $\:{M}_{S}$ encodes the locations to emphasize or suppress within the input feature map. This map is element-wise multiplied with the input tensor to highlight or diminish specific spatial locations based on the generated attention map. The process involves reducing the input tensor’s mean and max values across channels, concatenating these pooled features, and applying a convolution operation to obtain the attention map. Finally, this map is used to reweight the input tensor, enhancing the network’s focus on important spatial features. This attention mechanism effectively captures and emphasizes relevant spatial information, improving the model’s performance in tasks involving spatial relationships.

#### Channel attention

Channel attention mechanisms^38^ play a crucial role in enhancing the performance of CNNs by dynamically emphasizing informative features. The implemented Channel Attention class^39^ exemplifies this concept, utilizing both average and max pooling operations to capture comprehensive channel-wise information. Given an input feature map $\:X\:\in\:{R}^{(C\times\:H\times\:W)}$, where C, H, and W represent the number of channels, height, and width respectively, the mechanism begins by applying global average pooling ($\:F\_avg$) and max pooling $\:(F\_max)$ operations (11), (12).

11 $$F\_avg~ = ~GAP\left( X \right)$$

12 $$F\_max~ = ~GMP$$

where $\:GAP$ and $\:GMP$ denote global average pooling and global max pooling, respectively.

These pooled features are then processed through a shared multi-layer perceptron (MLP) as depicted in (13)

13 $$\:M\left(F\right)\:=\:\sigma\:\left({W}_{2}\:\delta\:\right({W}_{1}F\left)\right)$$

Here, $\:{W}_{1}\:\in\:\:{R}^{(C/r\times\:C)}$ and $\:{W}_{2}\:\in\:\:{R}^{(C\times\:C/r)}$ are the weights of the two dense layers, r is the reduction ratio, δ represents the ReLU activation function, and σ denotes the sigmoid function. The channel attention weights are computed by combining the MLP (Multi Layer Perceptron) outputs are depicted using (14)

14 $$\:M\:=\:\sigma\:\left(M\right(F\_avg)\:+\:M(F\_max\left)\right)$$

Finally, the attended feature map X’ is obtained by: (depicted by Eq. 15)

15 $$\:X{\prime\:}\:=\:M\:\odot\:\:X$$

where ⊙ represents element-wise multiplication.

This mechanism allows the network to learn complex channel interdependencies and focus on the most informative features. By returning both the attended features^40^ X’ and the attention weights M, it not only enhances performance but also provides valuable insights into the model’s feature prioritization process.

#### Temporal attention

Temporal attention mechanisms^41^ play a vital role in sequence modeling tasks by allowing neural networks to focus on relevant time steps dynamically. The implemented Temporal Attention class^42^ demonstrates this concept, employing a trainable context vector to compute attention weights across the temporal dimension.

Given an input sequence $\:X\:\in\:{R}^{T\times\:D},$ where T represents the number of time steps and D the feature dimensionality, the mechanism first projects the input into a hidden representation:

16 $$\:{u}_{it}\:=\:tanh(XW\:+\:b)$$

Here, $\:W\:\in\:{R}^{D\times\:U}$ is a weight matrix, $\:b\:\in\:\:{R}^{U}$ is a bias vector, and U is the number of units in the attention layer.

The attention weights are then computed using a context vector $\:u\:\in\:\:{R}^{U}$:

17 $$\:{a}_{it}=\:softmax\left({u}_{it}\:u\right)$$

where $\:{a}_{it}$ represents the importance of each time step.

The attended representation is obtained by weighting the input:

18 $$\:X{\prime\:}\:=\:\sum\:t\:{a}_{it}\:{X}_{t}$$

This mechanism allows the network to assign varying importance to different time steps, potentially capturing long-range dependencies more effectively than standard recurrent architectures.

By returning both the attended output X’ and the attention weights $\:{a}_{it}$, this approach not only enhances the model’s ability to focus on relevant temporal information but also provides interpretability, allowing researchers to analyze which time steps the model deems most crucial for its predictions.

The use of tanh activation^43^ in the hidden representation and softmax for attention weight normalization ensures bounded and properly distributed attention, contributing to stable training and effective temporal feature extraction.

### Dataset description

The effectiveness of any machine learning framework largely depends on the quality and diversity of the data used for training and testing. For this study, a comprehensive dataset of skin lesion images is utilized to develop and validate the proposed model. The original HAM10000 dataset images were collected across different dermatoscopic images from different populations, skin tones, acquired and stored by different modalities and with uniform image resolution across all images^58^. (Table 2).

The dataset used in this work is a balanced version^44^ of the original HAM 10,000 dataset and comprises a total of 32,851 training images, which include a diverse range of skin lesions classified into seven categories as described in Table 3. The images were sourced from well-established medical image repositories. As such, the dataset does not have any class imbalance. The dataset is split into training, validation, and testing sets to ensure robust model development and unbiased performance evaluation as in Table 2.

The comprehensive nature of the dataset, with a substantial number of images for each skin lesion category, enhances the reliability of the classification results produced by the proposed attention-based CNN-BiLSTM hybrid model. The detailed breakdown of the data sets ensures the model is thoroughly trained and validated, thereby improving its accuracy and generalizability. The data set was taken from^44^.

**Table 2:** Number of images in training, validation and testing dataset.

S. No.	Image type	Image count
1	Training	32,851
2	Validation	4697
3	Testing	9387

**Table 3:**
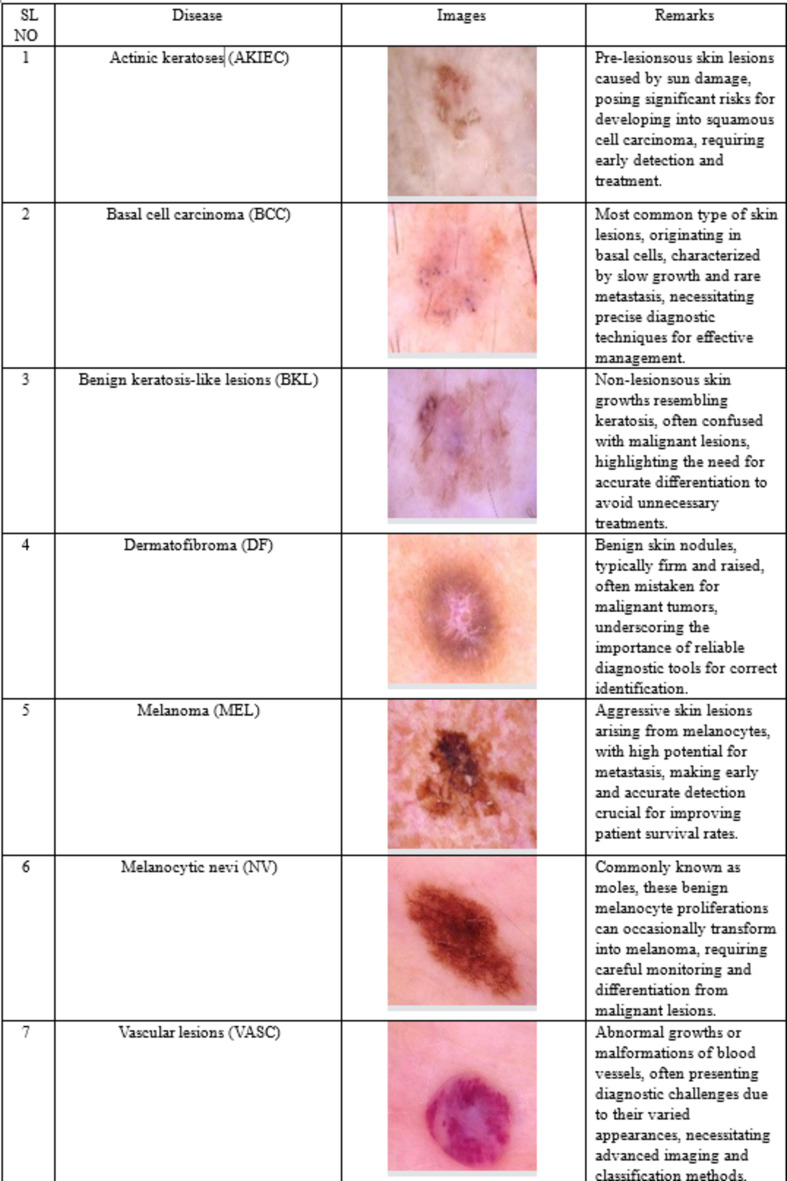
Description of the skin lesions in the dataset.

### Data augmentation

Data augmentation^45–47^ is a crucial technique for improving the performance and generalization of DL models by artificially increasing the size and variability of the training dataset. In this study, data was augmented by using the ImageDataGenerator class. The Table 4 below describes the transformations applied to the training and validation datasets for data augmentation, along with their corresponding parameters.

The data’s pixel values was rescaled by dividing them by 255 to standardize the pixel intensities between 0 and 1. Such normalization steps ensure that the model retains consistency and compatibility across all stages and also enhances computational efficiency. The proposed method does not include a denoising step, as different denoising algorithms can lead to significant information loss if not applied carefully.

**Table 4:** Data augmenting techniques used.

Transformation	Description	Parameters
Rescaling	Scales pixel values to a range between 0 and 1.	Rescale = 1/255
Rotation	Rotates the image by a random degree within a specified range.	Rotation_range = 20 (degrees)
Width Shift	Shifts the image horizontally by a fraction of the total width.	Width_shift_range = 0.1
Height Shift	Shifts the image vertically by a fraction of the total height.	Height_shift_range = 0.1
Zoom	Zooms in or out of the image within a specified range.	Zoom_range = 0.1
Shear	Applies a shear transformation to the image.	Shear_range = 10 (degrees)
Horizontal Flip	Randomly flips the image horizontally.	Horizontal_flip = True
Fill Mode	Fills gaps caused by transformations using the nearest pixel values.	Fill_mode=’nearest’
Validation Rescaling	Standardizes pixel values for validation data by dividing by 255.	Rescale = 1/255

Moreover, the model employs attention mechanisms to highlight important areas, which helps in reducing noise without significant information loss. Data augmentation techniques enhance the diversity and variance in the training dataset, allowing the model to learn from a broader range of image transformations and distortions. By training the model with augmented images, it becomes more resilient and better prepared to manage various lighting conditions, orientations, and distortions.

The images were resized to 224 × 224 pixels, processed in batches of 32, and labeled using categorical class mode with shuffling enabled to ensure random sampling.

### Proposed model

**Fig. 3:**
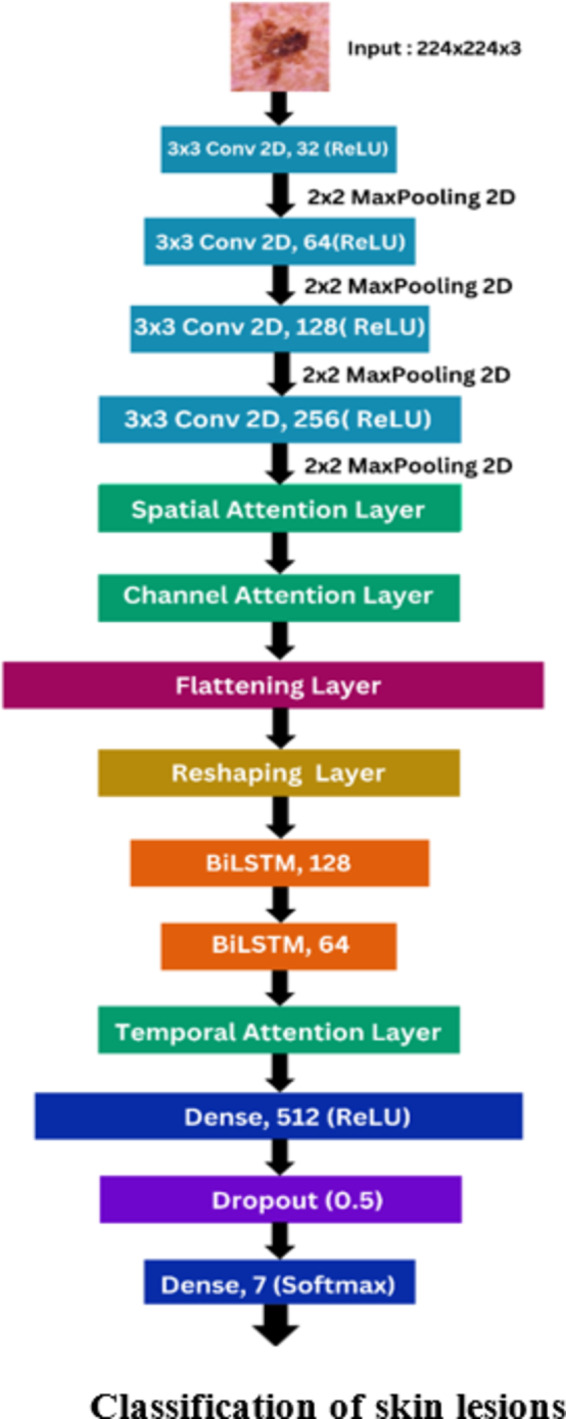
Model architecture of the proposed model.

The proposed model architecture is illustrated in Fig. 3. The proposed model integrates CNNs with BiLSTMs (refer Section III.B.1) and incorporates custom spatial, channel, and temporal attention mechanisms to effectively capture spatial, channel-wise, and temporal features from medical images, enhancing the robustness and reliability of disease diagnosis. The model begins with a series of convolutional and max-pooling layers, which are essential for extracting spatial features from the input images. The input layer accepts images of size 224 × 224 × 3, which corresponds to the dimensions and color channels of the images. The initial layers consist of Convolutional 2D (Conv2D) Layer 1 with 32 filters and a kernel size of 3 × 3, using the Rectified Linear Unit (ReLU) activation function.

The ReLU^48^ is described by the Eq. (19).

19 $$\:ReLU\left(x\right)=\:max\left(0,x\right)\:$$

This is followed by a MaxPooling 2D layer that reduces the spatial dimensions, aiding in the extraction of low-level features like edges and textures. This pattern continues with Conv2D Layer 2, which has 64 filters, and subsequent max-pooling. Conv2D Layer 3 and Layer 4 increase the filter size to 128 and 256 respectively, progressively capturing more complex features as the layers deepen. The architecture of the CNN layers has been already discussed in the custom CNN model (refer Section III.A).

**Fig. 4:**
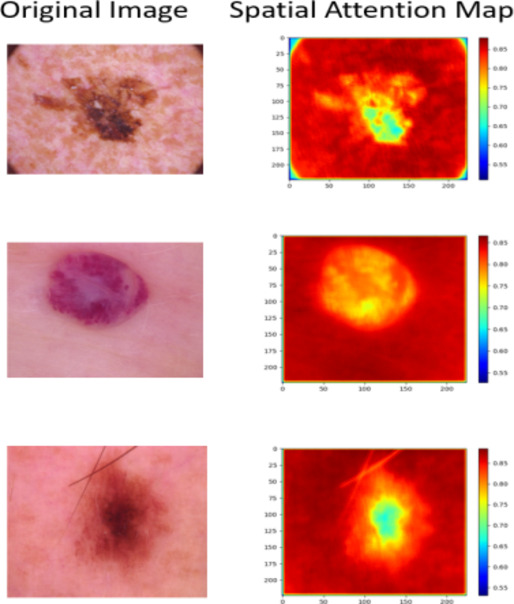
Original images and their spatial attention maps.

Custom layers for spatial, channel, and temporal attention are defined and utilized in the model. The spatial attention mechanism enhances spatial features by focusing on important regions within the images. A sample of images and their Spatial Attention maps is given Fig. 4. The channel attention mechanism assigns importance to different feature maps or channels, identifying the most critical aspects of the data. This is achieved by performing global average and max pooling operations, passing the results through shared dense layers, and combining the outputs to form a channel-wise attention map. The temporal attention mechanism is particularly useful for sequence data, enabling the model to focus on significant time steps within the sequences. It uses a dense layer to compute attention scores, which are then used to weigh the input features.

Following the CNN layers, the model reshapes the extracted feature maps into a sequence format to be fed into the BiLSTM layers. This transformation is achieved using a Reshape layer, which converts the 2D spatial features into a 1D sequence. The BiLSTM layers then process this sequence data to capture temporal dependencies and patterns. The use of BiLSTMs is particularly beneficial for skin lesion classification as it allows the model to maintain and leverage contextual information across the spatial dimensions of the image. BiLSTMs, a type of RNN, are capable of learning long-term dependencies and patterns within sequences. This property enables the model to better understand and classify complex patterns in skin lesions, which may involve subtle variations in texture, color, and structure over different regions of the image.

The final layers of the model consist of a Dense layer with 512 units and ReLU activation, followed by a Dropout layer. The output layer uses a softmax activation function to produce probability distributions for each class, with the number of units equal to the number of classes in the dataset.

The softmax activation function^49^ can be represented by the Eq. (20).

20 $$\:\sigma\:{\left({z}^{\to\:}\right)}_{i}=\frac{{e}^{{z}_{i}}}{{\sum\:}_{j=1}^{7}{e}^{{z}_{j}}}$$

Where$\:\:\sigma\:\:$is the softmax function, $\:{z}^{\to\:}\:is\:the\:$input vector, $\:{e}^{{z}_{i}}$ is the standard exponential function of the input tensor. The upper limit of the summation is 7 because of the 7 number of classes. $\:{e}^{{z}_{j}}$ is the standard exponential function of the output tensor.

#### Model training

The model is compiled using the Adam optimizer^50^ and categorical cross-entropy loss function, with accuracy as the performance metric. During training, several callbacks are employed to optimize the learning process. The ReduceLROnPlateau callback monitors the validation loss and reduces the learning rate by a factor of 0.2 if the validation loss plateaus for five epochs, with a minimum learning rate of 0.001. The EarlyStopping callback monitors the validation loss and stops training if the loss does not improve for ten consecutive epochs, restoring the best weights from the training process to prevent overfitting. Table 5 gives more details.

**Table 5:** Hyperparameter description.

Hyperparameter	Value	Description
Optimizer	Adam	Optimizer used for the training process.
Loss function	Categorical Cross-Entropy	Loss function to evaluate the model’s predictions against the true labels.
Performance metric	Accuracy	Metric used to assess the model’s performance during training.
Learning rate	Initial or Adaptive	Starting learning rate, adjusted by callbacks during training.
ReduceLROnPlateau factor	0.2	Factor by which the learning rate is reduced when validation loss plateaus.
ReduceLROnPlateau patience	5	Number of epochs with no improvement after which learning rate will be reduced.
ReduceLROnPlateau Min LR	0.001	Minimum learning rate after which it will not reduce further.
EarlyStopping patience	10	Number of epochs with no improvement after which training will be stopped.
EarlyStopping best weights	True	Option to restore the best weights from the training process to prevent overfitting.

The model is then trained on the augmented training data, with the validation data used to monitor its performance and adjust the learning process through the callbacks.

#### Model evaluation

Metrics^51–53^ from equations (21) to (28) such as Precision, Sensitivity (Recall), Accuracy, F1-score, Jaccard Index (JAC), Dice Coefficient (DIC), Matthews Correlation Coefficient (MCC) and Specificity were used to assess the model’s performance. In addition to these metrics we also used Area Under the Curve (AUC) score for validating the models. Below TP stands for True Positives, TN stands for True Negatives, FN stands for False Negatives and FP stands for False Positives.

21 $$\:\text{P}\text{r}\text{e}\text{c}\text{i}\text{s}\text{i}\text{o}\text{n}\:=\:\frac{TP}{TP+FP}$$

22 $$\:Sensitivity\:\left(Recall\right)=\:\frac{TP}{TP+FN}$$

23 $$\:Accuracy=\frac{TP+TN}{FP+FN+TP+TN}\:\:$$

24 $$\:F1Score=\:\frac{2\times\:Precision\times\:Recall}{Precision\:+\:Recall}$$

25 $$\:JAC=\:\frac{TP}{TP+FN+FP}$$

26 $$\:DIC=\frac{2\times\:TP}{(2\times\:TP)+FP+FN}$$

27 $$\:MCC=\frac{(TP\times\:TN)-(FP\times\:FN)}{\sqrt{(TP+FP)(TP+FN)(TN+FP)(TN+FN)}}$$

28 $$\:Specificity=\frac{TN}{TN+FP}$$

**Table 6:** Numerical comparison of evaluation metrics.

Model Name	Accuracy (%)	Precision (%)	F1 Score (%)	Sensitivity (Recall) (%)	Overall JAC score (%)	Overall DIC Score (%)	Overall MCC Score (%)	Specificity (%)	AUC (%)
CNN + BiLSTM + Attention Mechansims	**92.73**	**92.84**	**92.70**	**92.73**	**87.08**	**92.70**	**91.55**	**98.79**	**99.42**
CNN + LSTM + Attention Mechanisms	92.08	92.27	92.08	92.08	86.04	92.08	90.80	98.68	99.32
CNN + BiGRU + Attention Mechanisms	90.66	90.80	90.68	90.66	83.71	90.68	89.12	98.44	99.00
CNN + GRU + Attention Mechanisms	90.21	90.18	90.12	90.21	82.93	90.12	88.60	98.63	99.00
CNN + BiLSTM	88.96	88.82	88.87	88.96	81.13	88.87	87.13	96.99 ​	97.57
CNN + LSTM	90.02	89.97	89.96	90.02	82.68	89.96	88.37	​98.34	99.00
CNN + BiGRU	88.94	89.02	88.90	88.94	81.09	88.90	87.13	96.46	97.47
CNN + GRU	86.98	87.48	87.04	86.98	78.37	87.04	84.99	97.83	98.57
CNN	80.06	81.93	80.85	80.06	72.96	84.35	60.60	96.45	88.26
InceptionV3	81.63	82.43	81.69	81.63	70.51	81.69	78.67	96.57	89.10
VGG16	73.79	74.17	73.69	73.79	59.72	73.69	69.52	94.51	84.15
Xception	81.50	81.57	81.21	81.50	69.63	81.20	78.50	96.37	88.94

Significant values are given in bold.

**Fig. 5:**
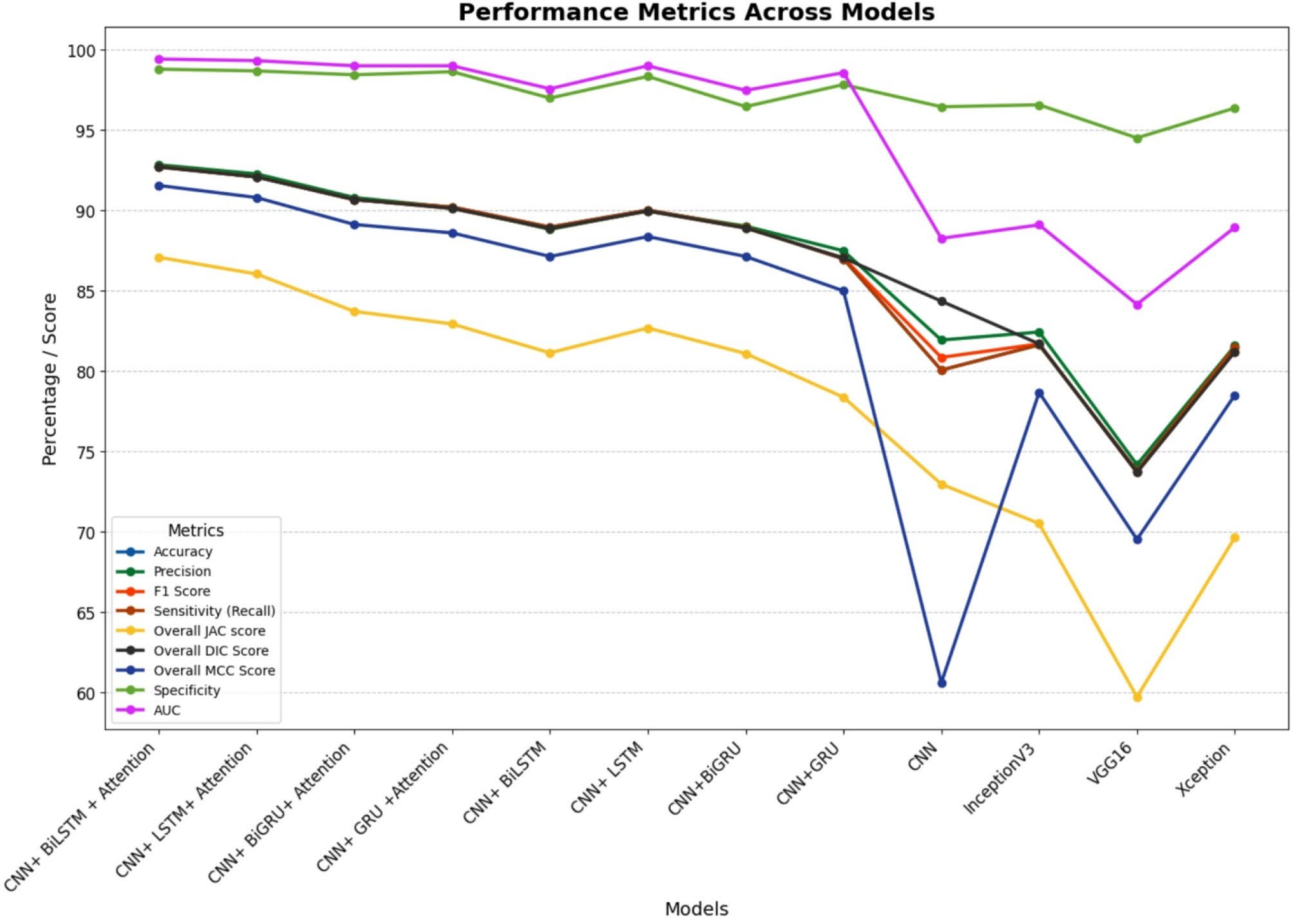
Comparison of performance metrics for various models.

**Fig. 6:**
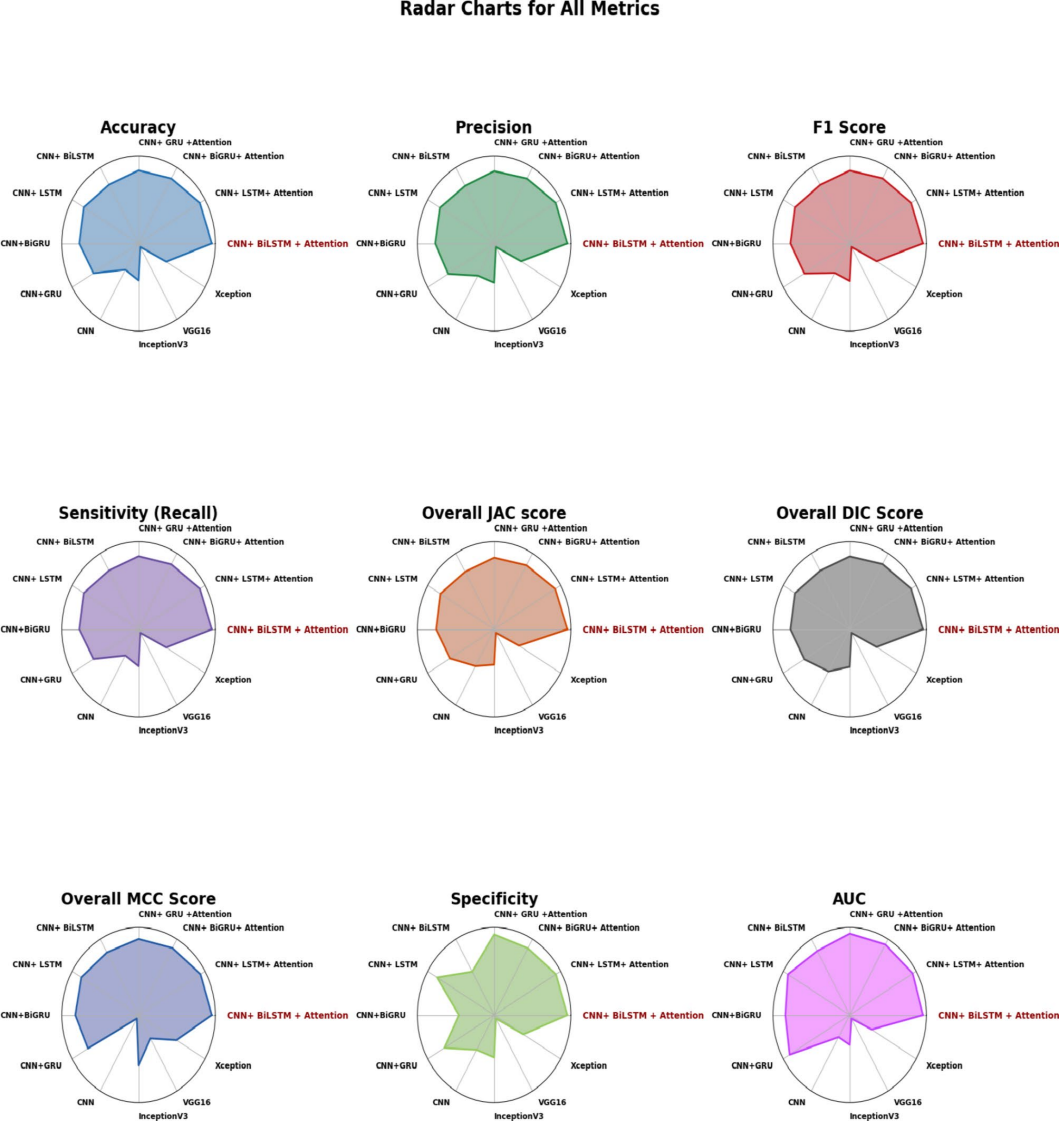
Radar chart depicting the performance of the proposed model.

## Experimental results and discussion

The Table 6; Fig. 5 provide a detailed evaluation of various deep learning models across multiple performance metrics, offering valuable insights into their strengths and weaknesses. Among these, the proposed model, CNN + BiLSTM with Attention Mechanisms, emerges as the clear leader, demonstrating superior performance in almost all metrics compared to its counterparts. With the highest accuracy of 92.73%, precision of 92.84%, F1 score of 92.70%, and sensitivity (recall) of 92.73%, it proves its ability to handle both false positives and false negatives effectively. Its unmatched specificity of 98.79% and AUC of 99.42% further reinforce its capability to distinguish between positive and negative classes with remarkable precision. Secondary metrics such as the JAC score (87.08%), DIC score (92.70%), and MCC score (91.55%) also highlight its well-rounded and robust performance, making it the most reliable model in this comparative study.

In comparison, other attention-based models, while performing admirably, fall short of the proposed model’s capabilities. The CNN + LSTM with Attention Mechanisms achieves high scores, such as 92.08% accuracy, 92.27% precision, and 92.08% F1 score, but consistently lags behind the proposed model in crucial metrics like JAC (86.04%) and MCC (90.80%). Similarly, the CNN + BiGRU with Attention Mechanisms, while competitive with an accuracy of 90.66% and precision of 90.80%, is unable to match the robustness of the proposed model. Its JAC score of 83.71% and MCC score of 89.12% reveal that it struggles to generalize as effectively, underlining the benefits of BiLSTM over GRU in capturing complex bidirectional dependencies. The CNN + GRU with Attention Mechanisms, although achieving respectable metrics such as 90.21% accuracy and 90.18% precision, performs significantly lower on JAC (82.93%) and MCC (88.60%), indicating its relative inferiority when compared to models with BiLSTM layers.

Non-attention-based models show a marked decline in performance, highlighting the critical role of attention mechanisms in enhancing model effectiveness. The CNN + BiLSTM achieves 88.96% accuracy and 88.82% precision, benefiting from the BiLSTM’s ability to capture sequential dependencies. However, the absence of an attention mechanism restricts its ability to focus on the most important features, resulting in lower scores such as a JAC score of 81.13% and MCC score of 87.13%. The CNN + LSTM and CNN + BiGRU models exhibit similar limitations, with accuracies of 90.02% and 88.94%, respectively, but lower scores across secondary metrics. Notably, the CNN + GRU performs significantly worse, with an accuracy of 86.98% and a steep drop in JAC (78.37%) and MCC (84.99%), making it less effective in capturing intricate relationships in the data.

The purely convolutional models, such as CNN, perform even worse. With an accuracy of 80.06% and an F1 score of 80.85%, it is clear that the lack of recurrent layers limits its ability to learn temporal and sequential patterns. Other pre-trained models, such as InceptionV3, VGG16, and Xception, also show subpar performance in comparison. For instance, InceptionV3 achieves an accuracy of 81.63% and a JAC score of 70.51%, while VGG16 lags further with only 73.79% accuracy and the lowest JAC score of 59.72%. These results underscore the limitations of these architectures for the given task, as they fail to match the performance of hybrid models combining CNNs with RNNs and attention mechanisms.

Overall, the CNN + BiLSTM with Attention Mechanisms consistently outperforms its competitors across all metrics, setting a benchmark for accuracy, precision, and overall reliability. Its ability to integrate the strengths of convolutional layers, bidirectional LSTMs, and attention mechanisms enables it to capture complex patterns and focus on critical features, making it the most effective and robust model in this evaluation. This comparative analysis clearly illustrates the superiority of the proposed model and its ability to deliver state-of-the-art performance for this task.

The radar charts in Fig. 6 effectively visualize the performance of various models across multiple metrics, highlighting the strengths of each. Among all the models compared, the proposed CNN + BiLSTM with Attention Mechanism stands out consistently, outperforming all other architectures across nearly every metric. Its near-perfect scores in Accuracy, Precision, and F1 Score demonstrate its ability to classify with exceptional reliability while maintaining a robust balance between true positive and true negative predictions. The superior Sensitivity and Specificity values reveal its capability to minimize both false negatives and false positives, making it an ideal model for scenarios where such balance is critical. Furthermore, its high Overall JAC, DIC, and MCC scores reinforce its ability to generalize effectively across diverse datasets, a feature that is less prominent in other models.

When compared to other attention-based models like CNN + LSTM with Attention or CNN + BiGRU with Attention, the proposed model not only leads in primary metrics but also excels in advanced indicators like AUC, which underscores its remarkable capability to distinguish between classes even in complex datasets. Purely convolutional models and pre-trained architectures such as VGG16, InceptionV3, and Xception exhibit a significant gap in performance, especially in metrics like JAC and MCC, reaffirming the superiority of hybrid designs that integrate convolutional layers with recurrent components and attention mechanisms.

Overall, the radar charts vividly showcase the dominance of the CNN + BiLSTM with Attention Mechanism, reflecting its unparalleled ability to capture intricate patterns and focus on relevant features while minimizing errors. This model’s comprehensive and consistent outperformance across all metrics solidifies its position as the most efficient and reliable among the architectures evaluated.

**Fig. 7:**
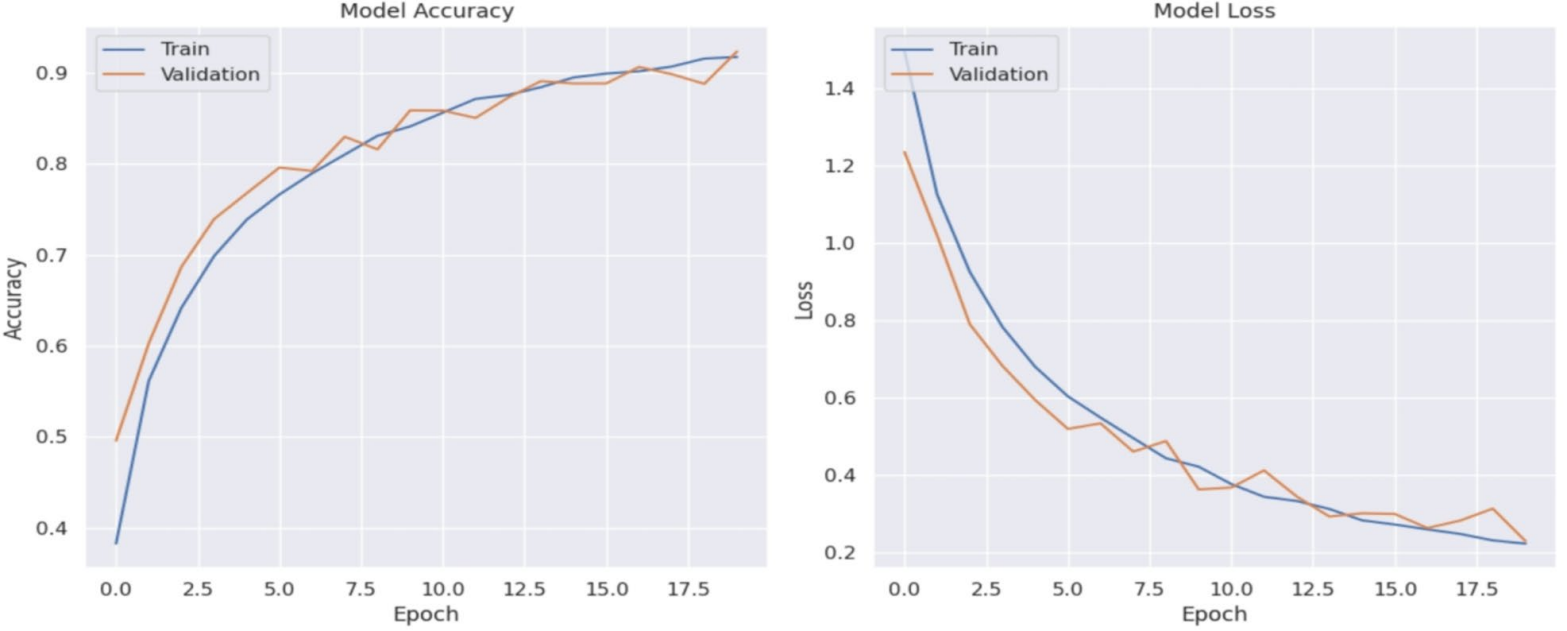
(**a**) Accuracy plot of the proposed model. (**b**) Loss plot of the proposed model.

The provided plots in Fig. 7(a), (b) illustrate the training and validation performance metrics for the CNN with BiLSTM and Attention Mechanisms model over 20 epochs, showcasing how the model’s accuracy and loss evolve during the training process.

The Fig. 7(a) shows the accuracy progression for both training and validation datasets. Initially, the model starts with an accuracy of approximately 40%. As training progresses, both training (blue line) and validation (orange line) accuracies show a steady upward trend. By the 20th epoch, the accuracies for both datasets exceed 90%. The close alignment of the training and validation accuracy curves indicates that the model is effectively learning from the training data while maintaining consistent performance on the validation data. This suggests that the model is not overfitting, as there is no significant divergence between the training and validation accuracy, a common issue where the model performs well on training data but poorly on unseen data.

The Fig. 7(b) illustrates the loss values for both training and validation datasets. Loss measures how well or poorly the model’s predictions match the actual results, with lowervalues indicating better performance. At the beginning of the training process, the loss for both training and validation is around 1.4. As training proceeds, there is a sharp decline in loss for both datasets, indicating rapid learning during the initial epochs. By the end of the training process, the loss values drop to approximately 0.2. Similar to the accuracy curves, the training loss (blue line) and validation loss (orange line) closely track each other, which further indicates good generalization by the model. The parallel descent of both curves towards lower loss values without significant gaps demonstrates that the model is efficiently capturing the patterns in the data without overfitting.

Overall, the plots in Fig. 7(a), (b) demonstrate that the proposed model achieves high accuracy and low loss on both training and validation datasets. The model’s ability to maintain high accuracy and low loss across epochs for both datasets highlights its robust performance and effectiveness in the image classification task. The integration of BiLSTM and attention mechanisms with CNN allows the model to leverage both spatial and contextual information, leading to superior feature extraction and better generalization capabilities, as evidenced by these performance metrics.

**Fig. 8:**
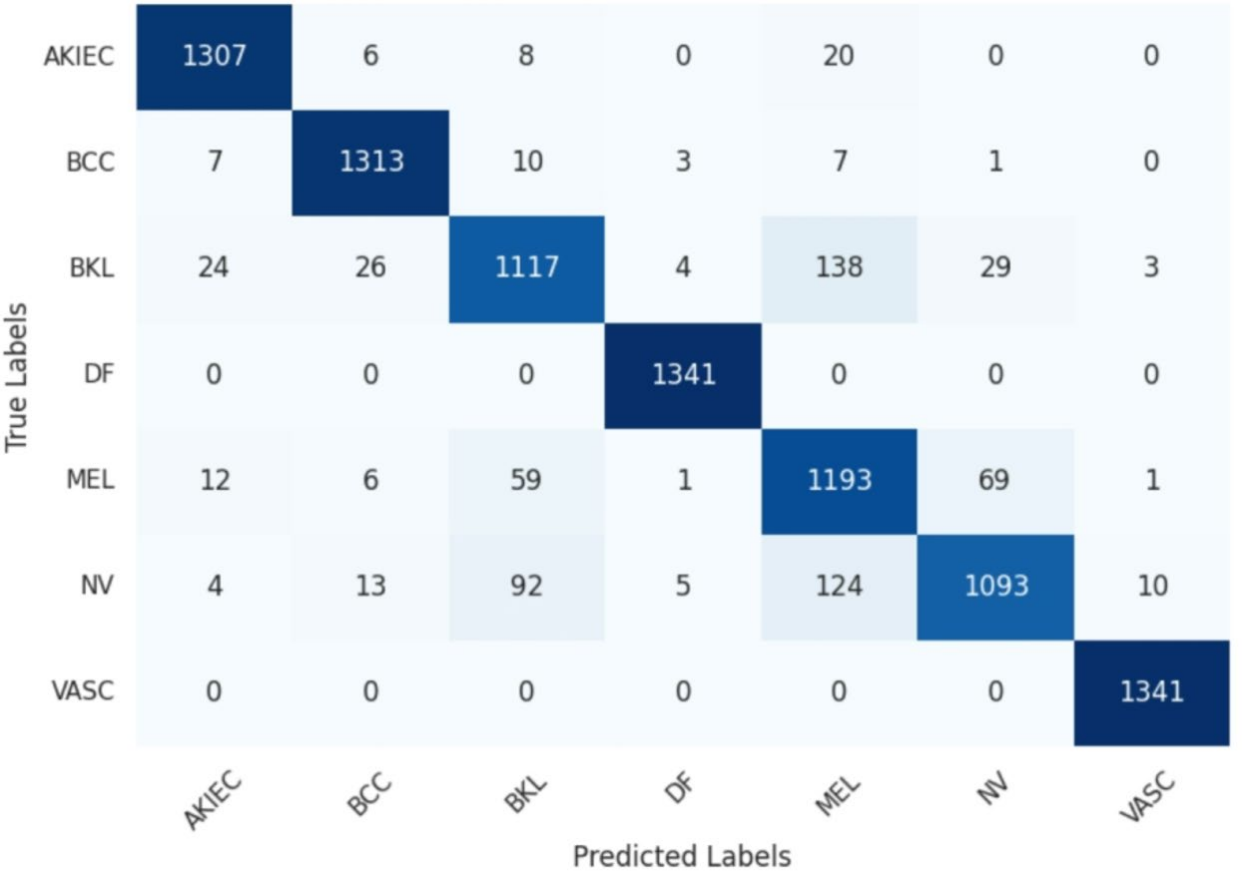
Confusion matrix achieved for the proposed model.

**Fig. 9:**
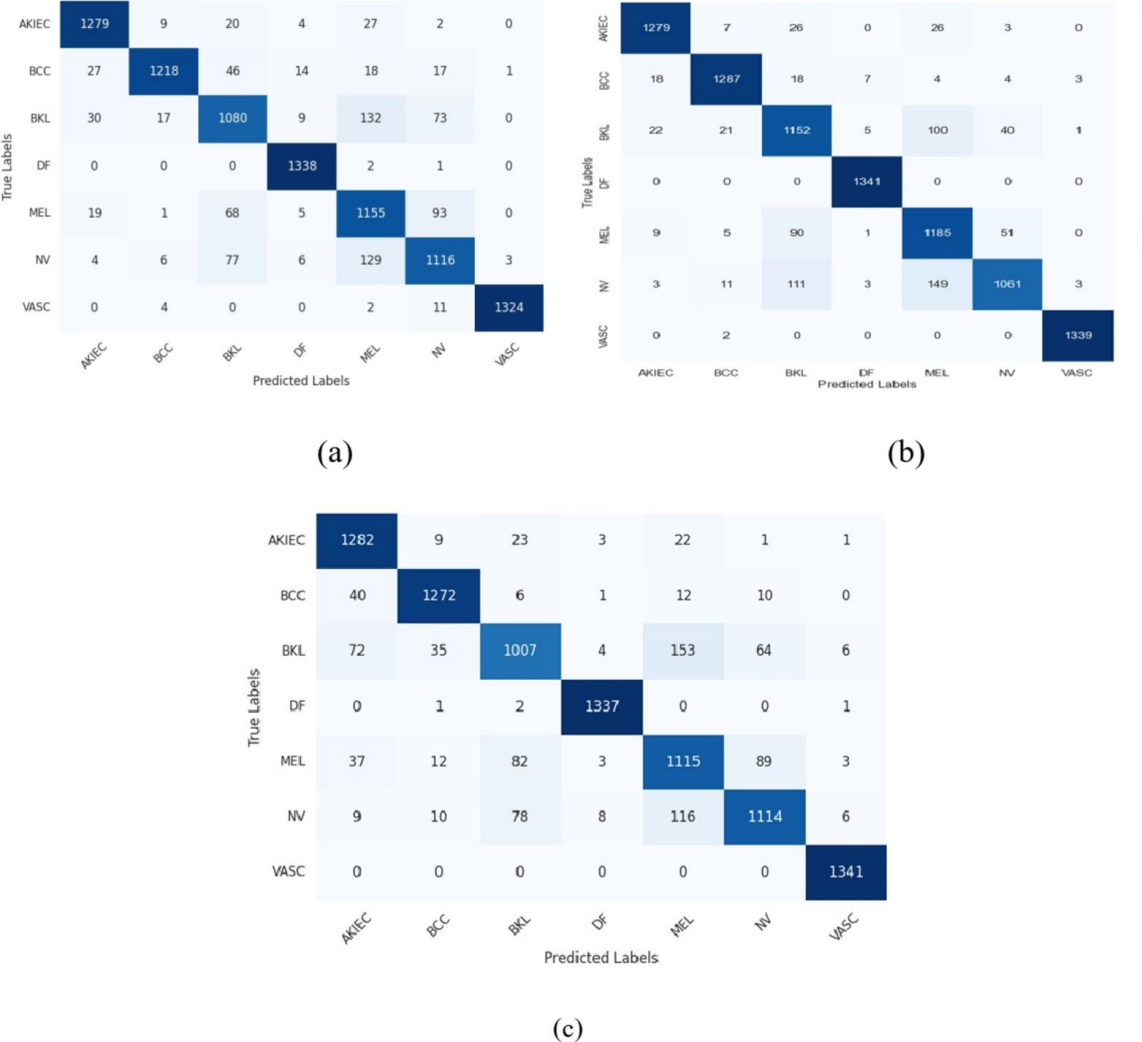
Confusion matrices for all other models with attention mechanisms. (**a**) CNN with BiGRU and attention mechanisms. (**b**) CNN with LSTM and attention mechanisms. (**c**) CNN with GRU and attention mechanisms.

In Fig. 8, the proposed model stands out as the top performer among the other three models depicted in Fig. 9. Its confusion matrix displays strong diagonal elements, indicating high classification accuracy across all seven classes. This model demonstrates a remarkable ability to distinguish between similar categories, with minimal off-diagonal elements suggesting low misclassification rates. The BiLSTM architecture appears to capture long-range dependencies in the data effectively, contributing to its superior performance.

The confusion matrix^54^ in Fig. 9(a), incorporating CNN with BiGRU and Attention Mechanisms, also shows impressive results, though with a slight increase in misclassifications compared to the BiLSTM model. This subtle difference suggests that while BiGRU is highly effective, the BiLSTM might have a marginal edge in capturing complex patterns within this particular dataset.

The confusion matrices in Fig. 9(b), (c), using CNN with LSTM and GRU respectively (both with Attention Mechanisms), demonstrate performance comparable to their bidirectional counterparts. However, they show minor decreases in accuracy for certain classes. This observation highlights the potential benefits of bidirectional processing in capturing context from both past and future states, which seems particularly advantageous for this classification task.

Across all four models, two classes consistently achieve near-perfect classification, suggesting these categories have highly distinctive features easily recognized by the neural networks. Conversely, two other classes show persistent confusion across all models, indicating a challenging similarity that even these sophisticated architectures struggle to fully differentiate.

The matrices reveal subtle but important differences in how each model handles the nuances of the classification task. For instance, the BiLSTM model appears particularly adept at correctly classifying two specific categories that the other models misclassify more frequently. This could indicate its superior ability to capture subtle, long-term dependencies crucial for distinguishing these particular classes.

In conclusion, while all four models demonstrate high proficiency in this complex classification task, the proposed model emerges as the frontrunner. Its marginally superior performance across all classes, especially in the more challenging categories, underscores the potential of this architectural combination for intricate image classification tasks. The consistent patterns of success and challenge across all models also provide valuable insights into the inherent difficulties of the dataset, potentially guiding future research directions in both model architecture and data preprocessing strategies.

**Fig. 10:**
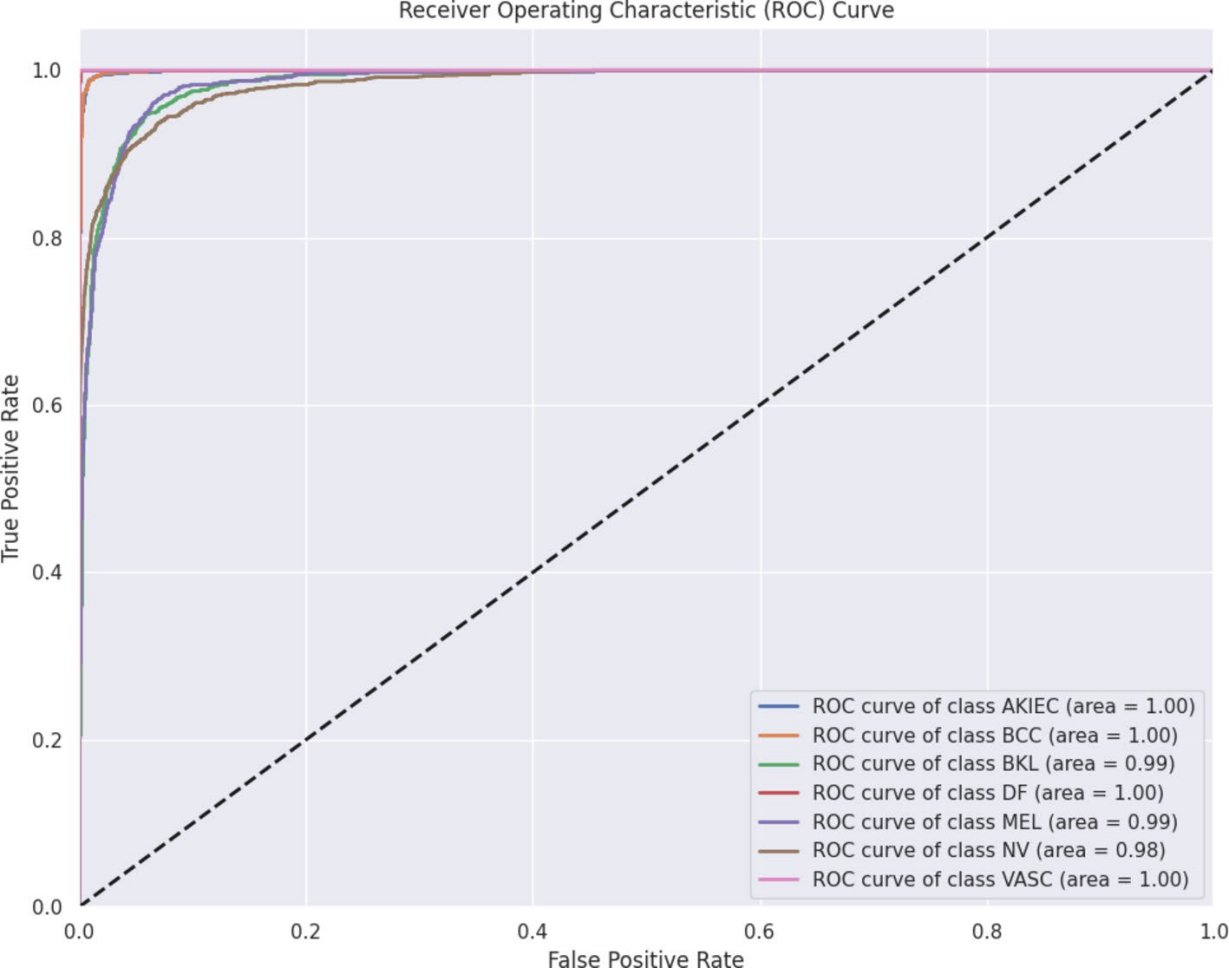
ROC curve for the proposed model.

Figure 10 presents a comprehensive Receiver Operating Characteristic (ROC)^55^ curve analysis for the proposed model. The graph illustrates the model’s exceptional performance across seven distinct classes: AKIEC, BCC, BKL, DF, MEL, NV, and VASC, each represented by a different coloured curve. All curves demonstrate remarkably high classification accuracy, hugging the top-left corner of the plot, which is indicative of an almost ideal classifier. The area under the curve (AUC) for each class ranges from 0.98 to 1.00, underscoring the model’s robust discriminative power. The diagonal dashed line, representing the performance of a random classifier, is far surpassed by all class curves, further emphasizing the model’s effectiveness. Notably, the curves for different classes are tightly clustered, suggesting consistent performance across all categories. This consistency is particularly impressive given the challenges often associated with multi-class medical image classification tasks. The proposed model’s ability to maintain such high accuracy across diverse classes showcases its potential as a powerful tool in automated medical diagnosis, potentially aiding in the early detection and classification of various skin conditions.

### Comparison with the state of Art works

In recent years, the field of automated skin lesion analysis has seen significant advancements driven by the integration of deep learning methods with computer vision techniques. A variety of approaches have been developed, each leveraging different neural network architectures, ensemble methods, and data augmentation techniques to improve diagnostic accuracy. The following Table 7 provides a detailed comparison of key metrics and methodologies from notable works, highlighting their unique contributions, strengths, and areas for improvement. This comparative analysis emphasizes the evolution of methods, tools, and performance measures across state-of-the-art studies.

**Table 7:** Comparison with the state of Art works.

Reference	Accuracy (%)	Sensitivity (%)	Specificity (%)	Methods and tools	Contribution
^25^	89.28	81	87.16	Inception-v3, ResNet-50, Inception-ResNet-v2, and DenseNet-201	Different CNN network integration for segmentation and multiple classification stages
^17^	87.42	97.04	97.23	ResNet152, InceptionResNet-V2, fine-tuning, Euclidean space, L-2 distance, transfer learning, Augmentation, GPU	Classify skin disease of faces using Euclidean space to compute L-2 distance between images
^26^	91	92	93	Inception V3 (GoogleNet), PECK, SCIDOG, SVM, RF, fine-tuning, transfer learning, Augmentation, GPU	Proposing an algorithm that is able to train CNN with limited data
^27^	87.7	85	73.29	AlexNet, VGG, ResNet-18, ResNet-101, SVM, MLP, random forest, transfer learning, Augmentation, GPU	Skin lesion classification using 4 CNNs and ensembling of the final classification results
Proposed Model	92.73	92.73	98.79	CNN with BiLSTM and attebntion mechanisms	Improved skin lesion classification

### Performance of our model on other datasets

To rigorously assess the generalizability and robustness of our proposed model, we validated its performance on two additional benchmark datasets, as referenced in^56,57^. For this evaluation, a carefully curated subset of 9,387 images was selected from each dataset, ensuring an equitable representation of 1,341 images per class to maintain balanced class distribution. The model consistently achieved outstanding results across datasets as shown in Table 8, across all key performance metrics, including accuracy, precision, recall, and specificity, alongside remarkable F1, JAC, DIC, and MCC scores. These results underscore the superior learning capability and discriminative power of the model, affirming its capacity to adapt to varying data distributions while preserving its predictive integrity. Such exemplary performance not only demonstrates the robustness of the model in diverse testing scenarios but also establishes its potential as a highly reliable tool for real-world applications, offering significant promise for deployment in challenging and dynamic environments.

**Table 8:** Performance of the proposed model on other datasets^56,57^.

Dataset	Accuracy (%)	Precision (%)	F1 score (%)	Sensitivity (recall) (%)	Overall JAC score (%)	Overall DIC Score (%)	Overall MCC Score (%)	Specificity (%)	AUC (%)
ISIC 2019^56^	93.97	94.05	93.95	93.97	89.11	93.97	93.01	98.99	99.56
ISIC 2018^57^	91.83	91.99	91.83	91.83	85.58	91.83	90.50	98.64	99.16

## Conclusion and future work

This work has introduced a pioneering model that integrates Convolutional Neural Networks (CNN) with Bidirectional Long Short-Term Memory (BiLSTM) units and Attention Mechanisms, demonstrating remarkable performance in medical diagnostics. This model achieves an impressive accuracy of 92.73%, with high precision, recall, and F1 scores, underscoring its robustness and reliability in classifying various skin conditions. The strength of this temporal patterns. The addition of attention mechanisms enhances the model’s capability to focus on the most critical aspects of the data, ensuring even subtle variations in medical images are meticulously examined. This approach paves the way for reliable and early detection of skin conditions. The custom CNN architecture, effectively captures the spatial features necessary for accurate diagnosis. However, further improvements to the CNN architecture itself could significantly enhance its performance when combined with recurrent neural networks (RNNs). By refining the CNN, the synergistic effect with RNNs can be amplified, leading to even more accurate and robust models.

The proposed model’s high performance, with an accuracy of 92.73%, precision of 92.84%, F1 score of 92.70%, recall of 92.73%, Jaccard Index (87.08%), Dice Coefficient (92.70%), and Matthews Correlation Coefficient (91.55%), underscores its potential to revolutionize clinical practices. High accuracy means the model correctly identifies most skin conditions. Precision shows that diagnoses are usually correct, minimizing false positives. The F1 score balances precision and recall, indicating overall reliability. Recall reflects the model’s ability to identify most actual cases, reducing false negatives. The Jaccard Index and Dice Coefficient measure how well predictions match real conditions, with higher values indicating better performance. The Matthews Correlation Coefficient provides a balanced measure of the model’s performance considering true positives, false positives, true negatives, and false negatives. Enhancements in data preprocessing, the exploration of additional hybrid models, and improvements in the CNN architecture could further elevate the capabilities of machine learning in medical imaging. Though the work provides robust results, there are a few challenges in implementing the same in real-world scenario. One of the major challenges could be the change in illumination while capturing the images. Another challenge could be the impact of heterogeneous background scenario while capturing the images that can dampen the accuracy. Future work could be focussed towards enhancing this model towards addressing these challenges using other vision and image processing techniques. Also, future work will be directed towards using this automated skin lesion classification model across patients with suitable guidance and support from medical professionals in this field.

In summary, this work not only presents a new model but also marks a significant step towards integrating advanced machine learning techniques into healthcare. This work promises a future where technology and medicine converge to save lives and enhance patient care, demonstrating the profound potential of machine learning in transforming medical diagnostics.

## Data Availability

The dataset used for this work is available in https://www.kaggle.com/datasets/shawon250/ham10000-balanced-128 × 128.
